# Genome-Annotated Bacterial Collection of the Barley Rhizosphere Microbiota

**DOI:** 10.1128/mra.01064-21

**Published:** 2022-02-17

**Authors:** Senga Robertson-Albertyn, James C. Abbott, Federico Concas, Lynn H. Brown, Jamie N. Orr, Timothy S. George, Davide Bulgarelli

**Affiliations:** a Plant Sciences, School of Life Sciences, University of Dundee, Dundee, United Kingdom; b Computational Biology, School of Life Sciences, University of Dundee, Dundee, United Kingdom; c Cell and Molecular Sciences, The James Hutton Institute, Dundee, United Kingdom; d Ecological Sciences, The James Hutton Institute, Dundee, United Kingdom; Indiana University, Bloomington

## Abstract

A culture collection of 41 bacteria isolated from the rhizosphere of cultivated barley (Hordeum vulgare subsp. *vulgare*) is available at the Division of Plant Sciences, University of Dundee (UK). The data include information on genes putatively implicated in nitrogen fixation, HCN channels, phosphate solubilization, and linked whole-genome sequences.

## ANNOUNCEMENT

The microbial communities thriving at the root-soil interface, that is, the rhizosphere microbiota, represent an untapped resource of plant probiotic functions (1, 2). Bacterial members of the microbiota capable of enhancing a plant’s mineral uptake from soil and pathogen protection, namely, plant growth-promoting rhizobacteria (PGPRs), have gained prominence in both basic scientific and translational applications (3–5). As a resource for comparative investigations of the plant microbiota across host species, we present a collection of 41 bacterial strains encompassing 15 genera with the presence of at least 5 putative plant growth-promoting (PGP)-associated gene orthologs, including, *dinG*, *hcn*, *nif*, *pho*, and *pqq* (Table 1) (6).

**TABLE 1:** Taxonomic affiliation, genomic characteristics, and accession numbers of genomes of 41 individual bacterial isolates^*a*^ described in this study

Isolate	Bacterial species^*b*^	Genome size (bp)	No. of contigs	*N*_50_ (bp)	PGPR operon gene(s) identified					GC content (%)	ENA accession no.
					*pqq*	*dinG* ^*c*^	*pho*	*nif*	*hcn*		
Bi02	Plantibacter cousiniae	3,994,224	78	112,363			C,H,R,U			69.52	ERS5639569
Bi03	Microbacterium foliorum	3,548,807	95	63,748	C,D	+	D,H,R,U			67.79	ERS5639570
Bi04	*Chryseobacterium* sp.	5,066,124	34	280,276			A,D,H,R	A,U		36.22	ERS5639571
Bi05	Agrobacterium fabrum	5,161,912	21	417,533			E,H,T,R,U	A,S,U		59.17	ERS5639572
Bi06	Pseudomonas brassicacearum	6,570,464	168	68,008	B,C,D,E,F,H	+	D,H,R,U	A	A,B,C	60.84	ERS5639573
Bi08	Pseudomonas carnis	6,697,465	117	94,982	B,C,D,E,F	+	D,H,R,U	A		59.44	ERS5639574
Bi13	Erwinia aphidicola	4,939,014	42	383,165	B,C,D,E,F	+	A,C,E,H,R,U			56.33	ERS5639577
Bi26	*Arthrobacter* sp. Bi26	5,112,477	217	43,594	F	+	H,R,U			66.4	ERS5639578
Bi27	*Pedobacter* sp. Bi27	6,285,703	23	908,772			A,H,R,U	A,U		39.01	ERS5639579
Bi35	Stenotrophomonas lactitubi	4,595,590	106	67,604		+	A,C,D,H,Q,R,U			66.03	ERS5639581
Bi36	*Pedobacter* sp. Bi36	6,291,372	23	901,067			A,H,R	A, U		38.98	ERS5639582
Bi44	Microbacterium foliorum	3,535,885	57	117,718		+	H,R,U			68.79	ERS5639584
Bi45	Enterobacter ludwigii	4,753,187	20	697,319		+	A,E,H,R,U	J		54.61	ERS5639585
Bi46	Agrobacterium fabrum	5,161,469	20	379,239			E,H,R,T,U	A, L, S, U		59.17	ERS5639586
Bi64	Pseudomonas *carnis*	6,761,930	127	163,868	B,C,D,E,F	+	A,D,H,R,U	A, U		58.02	ERS5639588
Bi66	Stenotrophomonas lactitubi	4,755,071	138	114,998	F	+	A,C,D,H,Q,R,U			65.19	ERS5639589
Bi70	Pseudomonas sp. Bi70	5,263,541	236	33,755	B,C,D,E,F,H	+	H,R,U			62.36	ERS5639590
Bi76	Microbacterium oxydans	3,812,380	110	78,635						68.34	ERS5639593
Bi80	Bacillus frigoritolerans	5,107,215	1,016	7,008						40.48	ERS5639594
Bi81	Stenotrophomonas lactitubi	4,657,418	91	99,706						65.95	ERS5639595
Bi82	Priestia megaterium	5,805,678	65	369,445						37.9	ERS5639596
Bi83	*Arthrobacter* sp. Bi83	4,893,187	121	76,950						65.19	ERS5639597
Bi84	Peribacillus simplex	5,576,719	465	27,035						39.86	ERS5639598
Bi89	Pseudomonas koreensis	6,136,482	73	165,400						60.15	ERS5639600
Bi91	Rhodococcus fascians	5,679,420	54	276,010						64.41	ERS5639601
Bi96	*Peribacillus* sp. Bi96	5,513,925	75	186,917						39.53	ERS5639602
Bi98	*Microbacterium* sp. Bi98	3,651,233	25	247,909						67.94	ERS5639603
Bi102	*Stenotrophomonas lactitubi*	4,657,928	103	75,776		+	A,C,D,H,Q,R			65.92	ERS5639604
Bi106	Rahnella aquatilis	4,557,699	1,614	3,019						51.67	ERS5639653
Bi110	Pseudomonas *carnis*	6,696,265	62	160,812						59.44	ERS5639654
Bi111	Pseudomonas *carnis*	6,695,877	169	75,930						59.44	ERS5639655
Bi112	Pseudomonas mediterranea	6,109,875	377	28,693	B	+	R,U		A,B,C	61.4	ERS5639656
Bi118	*Massilia* sp. Bi118	5,786,026	48	291,100	L	+	A,D,H,R,U,X			65.3	ERS5639658
Bi121	*Microbacterium* sp. Bi121	3,088,809	20	392,560			H,R,U			67.75	ERS5639659
Bi122	*Stenotrophomonas lactitubi*	4,652,813	125	66,023		+	A,C,D,H,Q,R,U			65.96	ERS5639660
Bi123	Pseudomonas sp. Bi123	6,405,172	52	250,449	B,C,D,E,F,H	+	D,H,R,U		A,B,C	59.37	ERS5639661
Bi126	*Pedobacter* sp. Bi126	6,291,746	22	1,097,689			A,H,R	A, L, U		38.98	ERS5639662
Bi128	*Microbacterium* sp. Bi128	6,566,320	1,694	4,998		+	H,R,U			68.6	ERS6138326
Bi130	Pseudomonas sp. Bi130	6,574,124	54	239,349	B,C,D,E,F,H	+	D,H,R,U	A	A,B,C	59.41	ERS5640634
Bi133	*Peribacillus simplex*	5,310,979	214	42,292		+	A,B,D,H,Q,R,U	S, U		40.03	ERS5640636
Bi134	*Peribacillus* sp. Bi134	5,473,506	31	522,968		+	A,B,D,H,Q,R,U			40.2	ERS5640637

a ANI cutoff, 96%. Capital letters depict actual genes identified within the inspected metabolic processes.

b Strain taxonomy reflects the lowest and unique rank as defined by GTDBTK (v1.6.0) with data version r202.

c The identification in each bacterial genome is depicted by the plus sign.

Strains were isolated from the rhizosphere of cultivated barley (Hordeum vulgare L. subsp. *vulgare*), the fourth most cultivated cereal worldwide (7), which was grown in an agricultural soil used for previous barley-microbiota investigations (8, 9). Bacterial rhizosphere fractions were obtained by detaching the soil adhering the uppermost 6 cm of barley roots by vortexing in phosphate-buffered saline (PBS) buffer. Serial dilutions were plated onto R2A and nutrient agar media and incubated at 20°C for 48 to 72 h (10, 11). Individual CFUs were selected for isolation based on morphological variation; clean isolate liquid cultures were stored at −80°C in 70% glycerol following 24 to 48 h of shaking incubation at 27°C.

**FIG 1:**
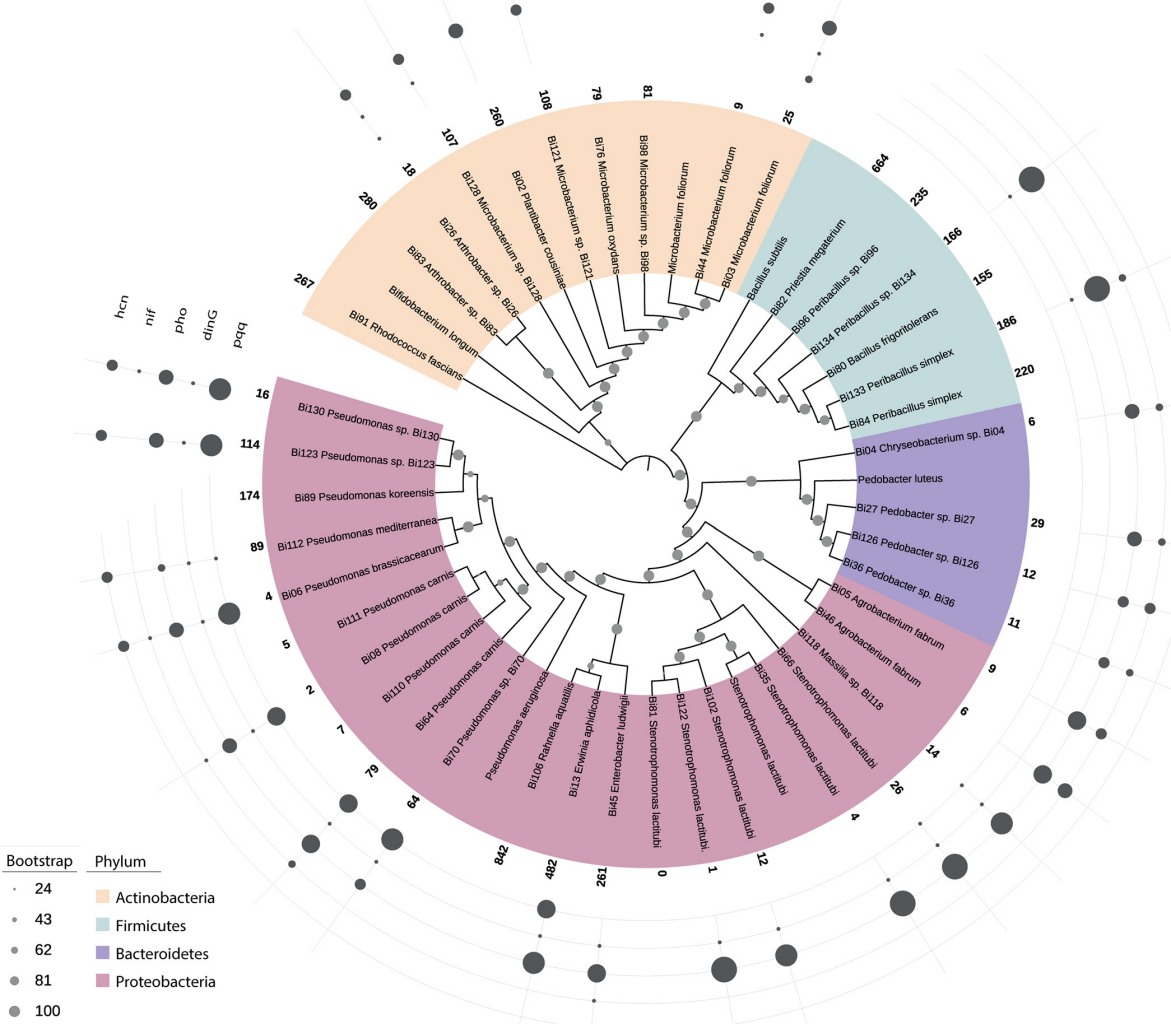
Whole-genome phylogenetic tree of individual genomes (ANI cutoff, 96%) constructed incorporating additional sequences for Bifidobacterium longum NCC2705 (GCA_000007525), Microbacterium foliorum DSM 12966 (GCA_000956415), Bacillus subtilis NCIB 3610 (GCA_006088795), Pedobacter luteus DSM 22385 (GCA_900168015), Stenotrophomonas lactitubi M15 (GCA_002803515), and Pseudomonas aeruginosa PAO1 (GCA_000006765). Protein predictions were obtained using Prokka (v1.14.6), and the tree was constructed with 100 bootstrap iterations and annotated with iTOL (24). The size of circular shapes on the periphery of the tree reflects the number of the indicated PGPR genes ranging from 1 to 7 present in each individual sample.

DNA was extracted as per the manufacturer’s instructions using the FastDNA spin kit for soil (MP Biomedicals, USA). Individual bacterial isolates were subjected to whole-genome sequencing using the “standard service” of MicrobesNG (Birmingham, UK). Briefly, bacterial genomic DNA libraries were prepared using the Nextera XT library prep kit (Illumina, USA) following the manufacturer’s protocol with the following modifications: 2 ng of DNA were input, and PCR elongation time was increased to 1 min. DNA quantification and library preparation were conducted on a Hamilton Microlab STAR automated liquid handling system. Pooled libraries were quantified using the Kapa Biosystems library quantification kit for Illumina on a Roche light cycler 96 quantitative PCR (qPCR) machine. Libraries were sequenced by using an Illumina HiSeq instrument with a 250-bp paired-end protocol. Reads were adapter trimmed using Trimmomatic (v0.30) with a sliding window quality cutoff of Q15 (12). *De novo* assembly was performed using SPAdes (v3.7), and contigs were annotated using Prokka (v1.12) (13, 14). On the basis of GC content, unambiguous taxonomic annotations generated using amphora classification (15) and whole-genome average nucleotide identity (ANI) to identify individual “founder” members (ANI cutoff, 96%) yielded 41 genomes retained for downstream analyses. To compare only components of characterized metabolic pathways, predicted genes were concatenated and annotated with eggNOG-Mapper (v1.0.3) (16, 17). The resultant annotation file was parsed in Python to generate a table of taxonomic identities (IDs) of Kyoto Encyclopedia of Genes and Genomes (KEGG) ortholog (KO) identifiers. From this table, a presence-absence matrix of all KOs predicted at least once in each isolate was generated in R (https://www.r-project.org). Predicted proteomes were clustered using OrthoFinder (v2.2.1) and functionally annotated using InterProScan (v5.29-68.0) (18, 19). Clusters and annotations were aggregated using KinFin (v1.0) (20). Cluster and KO intersections were defined using UpSetR (v1.3.3) (21). The phylogenetic tree (Fig. 1) was constructed using bcgTree (v1.1.0) and RAxML (v8.2.12), using RAxML’s GTRGAMMA model and 100 bootstrap iterations (22, 23); default parameters were used for all analyses unless otherwise noted.

The collection is available as frozen isolates preserved in 300 to 500 µL of nutrient or R2A medium containing 50% glycerol and maintained at −70°C. To revive the frozen cultures, we recommend using a sterile inoculating loop to transfer a small amount (e.g., 50 µL equivalent) of the frozen culture onto a nutrient or R2A agar medium base following standard microbiological procedures. The plates should be incubated at 27°C for 24 to 48 h.

### Data availability.

The genomic sequences reported in this study are deposited in the European Nucleotide Archive (ENA). Accession numbers for the individual genomes are provided in Table 1. To acquire isolates, or for questions or suggestions, please contact Davide Bulgarelli at d.bulgarelli@dundee.ac.uk.
